# Generation of a GFP Reporter Akabane Virus with Enhanced Fluorescence Intensity by Modification of Artificial Ambisense S Genome

**DOI:** 10.3390/v11070634

**Published:** 2019-07-10

**Authors:** Akiko Takenaka-Uema, Shin Murakami, Nanako Ushio, Tomoya Kobayashi-Kitamura, Masashi Uema, Kazuyuki Uchida, Taisuke Horimoto

**Affiliations:** 1 Department of Veterinary Microbiology, Graduate School of Agricultural and Life Sciences, The University of Tokyo, 1-1-1 Yayoi, Bunkyo-ku, Tokyo 113-8657, Japan; 2 Department of Veterinary Pathology, Graduate School of Agriculture and Life Sciences, The University of Tokyo, 1-1-1 Yayoi, Bunkyo-ku, Tokyo 113-8657, Japan; 3 Division of Biomedical Food Research, National Institute of Health Sciences, 3-25-26 Tonomachi, Kawasaki-ku, Kawasaki 210-9501, Japan

**Keywords:** Akabane virus, ambisense S genome, untranslated region, in vivo imaging

## Abstract

We previously generated a recombinant reporter Akabane virus expressing enhanced green fluorescence protein (eGFP-AKAV), with an artificial S genome encoding eGFP in the ambisense RNA. Although the eGFP-AKAV was able to detect infected cells in in vivo histopathological study, its fluorescent signal was too weak to apply to in vivo imaging study. Here, we successfully generated a modified reporter, eGFP/38-AKAV, with 38-nucleotide deletion of the internal region of the 5′ untranslated region of S RNA. The eGFP/38-AKAV expressed higher intensity of eGFP fluorescence both in vitro and in vivo than the original eGFP-AKAV did. In addition, eGFP/38-AKAV was pathogenic in mice at a comparable level to that in wild-type AKAV. In the mice infected with eGFP/38-AKAV, the fluorescent signals, i.e., the virus-infected cells, were detected in the central nervous system using the whole-organ imaging. Our findings indicate that eGFP/38-AKAV could be used as a powerful tool to help elucidate the dynamics of AKAV in vivo.

## 1. Introduction

Akabane virus (AKAV) belongs to the genus *Orthobunyavirus,* family *Peribunyaviridae,* and order *Bunyavirales* and is an arthropod-borne virus that causes a reproductive disorder known as “arthrogryposis-hydraencephaly syndrome,” which is characterized by abortion, stillbirth, premature birth, and congenital deformities in livestock, such as cattle, sheep, and goats.

Orthobunyaviruses, including AKAV, are comprised of genomes with three negative-sense RNA segments, denoted as large (L), medium (M), and small (S) segments. The L segment encodes RNA-dependent RNA polymerase. The M segment encodes enveloped glycoproteins Gn and Gc, which are responsible for cell attachment, and a nonstructural NSm protein, which likely affects viral replication [1]. The S segment encodes nucleocapsid (N) protein, which encapsidates the viral RNA in ribonucleoprotein (RNP) complex, and encodes a non-structural NSs protein, which suppresses the host-cell antiviral response [2]. The N and NSs proteins are encoded in overlapped open reading frames and translated from the same mRNA using different initiation codons [3]. The coding region of each segment is flanked by 3′ and 5′ untranslated regions (UTRs), which contain conserved 11-nucleotide (nt) sequences at each terminus of the three segments [4]. The genomic RNA of AKAV, as in other bunyaviruses, forms the panhandle structure at complementary sequences of the 3′ and 5′ ends [5,6,7,8], which plays important roles in genomic RNA replication, transcription, encapsidation, and packaging [9].

Unlike orthobunyaviruses, phleboviruses, such as Rift Valley fever virus (RVFV), contain ambisense S RNA, which carries two oppositely oriented N and NSs reading frames separated by an intergenic region (IGR). To transcribe the N and NSs gene, RVFV S RNA possesses a transcriptional promoter at both its 5′ and 3′ ends. Although orthobunyaviruses, such as AKAV and Bunyamwera virus (BUNV), do not use the ambisense strategy, the 5′ end of S RNA possesses weak transcriptional activity [10,11,12,13]. Based on this feature, we previously generated a recombinant reporter AKAV including enhanced green fluorescent protein (eGFP) gene by utilizing an ambisense strategy, so called eGFP-AKAV [12]. The eGFP-AKAV consists of wild-type L and M and an artificial ambisense S genome, which contains two oppositely oriented N/NSs (negative-sense) and eGFP (positive-sense) genes, separated by an IGR sequence of RVFV. Although the eGFP-AKAV stably expressed eGFP and facilitated the visualization of virus-infected cells in vitro, its fluorescent signal was too weak for in vivo imaging studies.

The 5′ UTR sequence of AKAV S RNA is much longer than RVFV S RNA (e.g., 121-nt vs. 34-nt). The long 5′ UTR sequence of orthobunyavirus S RNA contains cis-acting RNA replication promoter in at least 30-nt region at their terminus [11] and transcription termination signal, which located at the sequence region between the replicational promoter and ORF [12,13,14,15,16,17,18]. The transcription of N/NSs mRNA of wild-type AKAV terminated at the 5′ UTR, whereas in case of eGFP-AKAV, transcription termination of N/NSs and eGFP occurred at the inserted IGR [12], suggesting that an authentic transcription terminal signal is not required for expression of N/NSs mRNAs. The 5′ UTR of orthobunyavirus S RNA formed a hairpin structure at the transcription termination signal [18], which may inhibit transcription from the promoter of AKAV S 5′ UTR. Therefore, we postulate that partial deletions of 5′ UTR, including the transcription termination signal of the eGFP-S segment, may enhance eGFP expression without affecting viral replication.

In this study, we generated a series of eGFP-AKAV viruses with partial deletions of 5′ UTR of S RNA and evaluated their replication property and the intensity of their fluorescent signals using in vivo imaging in a mouse model.

## 2. Materials and Methods

### 2.1. Cells and Viruses

Baby hamster kidney cells stably expressing T7 RNA polymerase (BHK/T7-9 cells) [19] were kindly provided by Dr. Naoto Ito (Gifu University, Japan) and cultured at 37 °C in Eagle′s minimum essential medium (MEM) supplemented with 5% fetal bovine serum (FBS) and 10% tryptose phosphate broth. Hamster lung (HmLu-1) cells, obtained from National Institute of Animal Health, Japan, were cultured at 37 °C in Dulbecco’s modified Eagle’s medium (DMEM) supplemented with 5% FBS. We used eGFP-AKAV and AKAV-RG (wild-type) which were the neurovirulent Iriki strain [20]-based recombinant viruses generated by reverse genetics [12]. Viruses were propagated in HmLu-1 cells in serum-free medium.

### 2.2. Plasmid Construction

**Figure 1:**
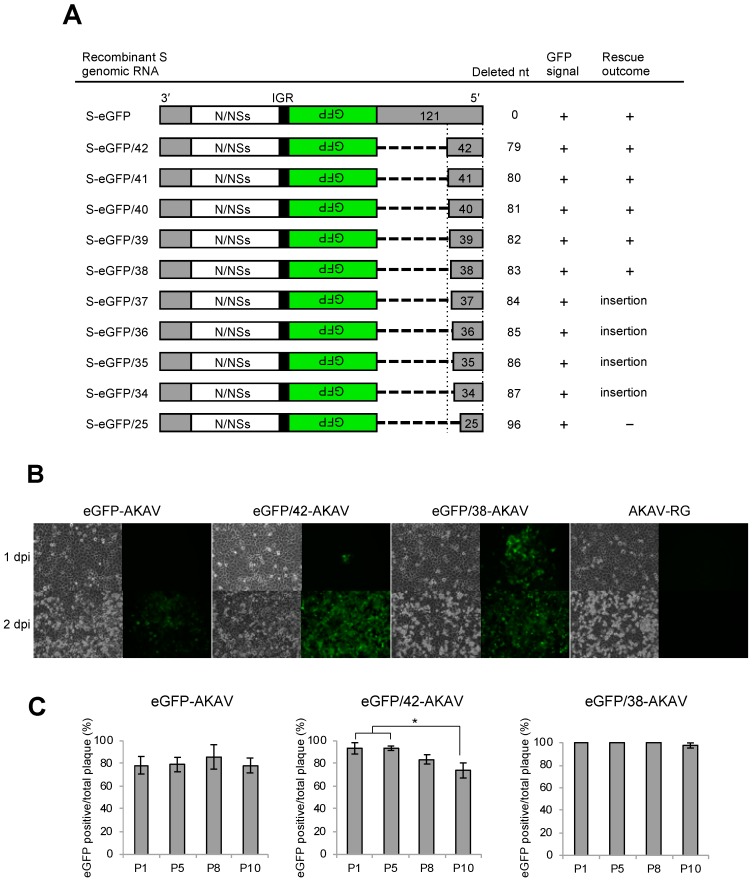
Generation and genetic stability of recombinant reporter Akabane viruses (AKAVs). (**A**) Schematic diagram of deletions introduced in the 5′ untranslated region (UTR) of the ambisense S segment are shown. Horizontal dotted lines indicate deleted regions. Gray bars represent the UTR on each side. White and green bars represent the nucleocapsid negative sense (N/NSs) and green fluorescent protein (GFP) coding regions, respectively. Black bars represent the intergenic region (IGR) of Rift Valley fever virus (RVFV). + denotes enhanced green fluorescent protein (eGFP) expression in transfected cells. - denotes no rescue of recombinant virus by reverse genetics. “insertion” denotes the presence of unexpected insertion sequences in the S 5′ UTR of the rescued viruses. (**B**) Cytopathic effects (CPE) and eGFP expression in cells infected with parent eGFP-AKAV, deletion viruses (eGFP/42- and eGFP/38-AKAVs), and wild-type AKAV-RG are shown. Panels on the left are images with bright field and panels on the right are fluorescence images. (**C**) To assess the genetic stability of eGFP-, eGFP/42-, and eGFP/38-AKAVs, they were passaged 10 times in HmLu-1 cells. At passages 1, 5, 8, and 10, viruses were subjected to a plaque assay. The numbers of total and eGFP-positive plaques were determined. The results are presented as the mean ± SD (error bars) from 4–5 wells. *, *p* < 0.01; Mann–Whitney *U* test.

Plasmid pT7riboSM2/S-eGFP [12] was derived from pT7riboSM2/IS where cDNAs from the S segment of the Iriki strain was cloned. S-eGFP was previously constructed as an ambisense S-segment, which contained an RVFV IGR sequence and the eGFP gene in the opposite orientation to the AKAV N/NSs genes, with intact 3′ and 5′ UTR sequences of the S segment (Figure 1A). To produce the deletion constructs of 5′ UTR, pT7riboSM2/S-eGFP was used as a template for PCR amplification. The PCR was performed with KOD-Plus-Neo (TOYOBO, Osaka, Japan) using primer pairs, T7riboSM2-SF [21] and S842-815/GFP (5′-CTAATTAACTATAAACAATAAAATCCAAATGGTGAGCAAGGGC-3′) for deletion of nucleotides (nt) 736-814 (42 nt left in the 5′ UTR; namely S-eGFP/42), S842-823/GFP (5′-CTAATTAACTATAAACAATAATGGTGAGCAAGGGC-3′) for deletion of nt 736-822 (34 nt left in the 5′ UTR; S-eGFP/34), or S856-832/GFP (5′-AGTAGTGTTCTCCACTAATTAACTAATGGTGAGCAAGGGC-3′) for deletion of nt 736-831 (25 nt left in the 5′ UTR; S-eGFP/25) of the AKAV S segment. Each PCR product was used as a template for sequential PCR amplification using the primers, T7robpSM2-SF, and T7riboSR-S828 (5′-AATCGTCTCCACCCAGTAGTGTTCTCCACTAATTAACTATAAA-3′) (S-eGFP/42 and S-eGFP/34), or T7riboSM2-SR2 [21] (S-eGFP/25). All 5′-truncated S-eGFP mutants were inserted into a pT7riboSM2 vector [22] at the Esp3I restriction site, yielding pT7riboSM2/S-eGFP/42, -/34, and -/25, as described previously [21]. Similarly, T7riboSM2/S-eGFP/41, -/40, -/39, -/38, -/37, -/36, and -/35 were generated using primer pairs, T7riboSM2-SF and S842-816/GFP, S842-817/GFP, S842-818/GFP, S842-819/GFP, S842-820/GFP, S842-821/GFP, or S842-822/GFP, respectively (primer sequences are provided upon request), and then T7riboSM2-SF and T7riboSR-S828. All constructs were confirmed by DNA sequencing using standard protocols and a 3130xl Genetic Analyzer (Life Technologies Japan, Applied Biosystems, Tokyo, Japan).

### 2.3. Reverse Genetics

To generate recombinant AKAVs using the T7 polymerase-dependent rescue system, subconfluent BHK/T7-9 cells were grown in six-well plates. The cells in each well were transfected with 1.2 µg of pT7riboSM2/IL and T7riboSM2/S-eGFP/42 to -/25, and 0.6 µg of pT7riboSM2/IM using 9 µL TransIT-LT1 transfection reagent (Mirus Bio, Madison, WI, USA) in 200 µL OPTI-MEM (Life Technologies/GIBCO, Grand Island, NY, USA), as described previously [21]. At 3 to 7 days post-transfection, once the cytopathic effects (CPE) were observed, the culture supernatants were harvested and added to HmLu-1 cells. The generation of recombinant viruses was confirmed by CPE and expression of eGFP under a fluorescent microscope (Axio Vert.A1, Zeiss, Oberkochen, Germany). All recombinant viruses were plaque-purified three times on HmLu-1 cells and stored for subsequent experiments. The titers of the recombinant viruses were determined using a plaque assay, as previously described [23].

All recombinant viruses were confirmed to possess the intended nucleotide deletions of their S segments by RT-PCR followed by sequencing. Viral RNAs were extracted from the supernatants of infected cell cultures using a QIAamp Viral RNA Mini Kit (QIAGEN, Tokyo, Japan), according to the manufacturer’s instructions. To synthesize the cDNAs, we used SuperScript^®^ III reverse transcriptase (Invitrogen, Thermo Fisher Scientific, Tokyo, Japan) and a primer SF (5′-AGT AGT GAA CTC CAC TAT TAA CTA CGC-3′). The S segments of the recombinant viruses were amplified by PCR using KOD FX Neo and primers SF and GFP-588F (5′-GCC CGA CAA CCA CTA CCT GA-3′), and GFP-R1 (5′-TTA CTT GTA CAG CTC GTC CAT G-3′) and SR (5′-AGT AGT GTT CTC CAC TAA TTA ACT ATA-3′), as two overlapping fragments. The former RT-PCR products were directly sequenced. The latter products were dA-attached using a 10× A-attachment mix (TOYOBO), cloned into pCR2.1-TOPO (Invitrogen), and sequenced using standard protocols.

### 2.4. Counting GFP-Positive Plaques

The recombinant deletion virus, eGFP/42-AKAV or eGFP/38-AKAV, was serially passaged 10 times in HmLu-1 cells. The cells, grown on a 4-well Lab-Tek chamber slide (Thermo Science Nunc, Tokyo, Japan), were infected with diluted deletion and parent eGFP-AKAVs at various passages for 1 h at 37 °C. After any unbound viruses were removed, the cells were covered with DMEM containing 0.8% methylcellulose and 2% FBS. At 3 days post-infection (dpi), the media were removed, and the cells were washed with phosphate-buffered saline (PBS) three times. Live cells were observed using a Zeiss LSM700 confocal microscope. The total plaques and fluorescent plaques were counted and compared at different points of the virus passages. Data were collected from at least four wells for each virus.

### 2.5. Growth Kinetics and Plaque Morphology

A subconfluent HmLu-1 cell monolayer was infected with either eGFP/42-AKAV, eGFP/38-AKAV, parent eGFP-AKAV, or wt AKAV-RG at a multiplicity of infection (MOI) of 0.01. After 1 h at 37 °C, any unbound virus was removed, cells were washed with PBS, and serum-free medium was added. At different time-points post-infection, the supernatants were harvested and titrated using the plaque assay with HmLu-1 cells. To examine the differences in plaque morphology between the deletion viruses, eGFP-AKAV, and AKAV-RG, HmLu-1 cells were seeded onto 6-well plates. The viruses were added to cells and incubated at 37 °C for 1 h. Then, any unbound viruses were removed and the cells were covered with DMEM containing 0.8% (*w/v*) agar and 2% FBS. After 4 days, the cells were stained with neutral red.

### 2.6. Immunofluorescence Assays

Subconfluent HmLu-1 cell monolayers on 4-well chamber slides were infected with recombinant viruses, as well as the parent eGFP-AKAV and wt AKAV-RG viruses, at a MOI of 0.01 for 1 h at 37 °C. After the removal of unbound viruses, the cells were overlaid with DMEM containing 0.8% methylcellulose and 2% FBS. At 3 dpi, the media were removed, and the cells were washed with PBS. Cells were fixed with 4% paraformaldehyde and permeabilized with 0.3% Triton X-100 in PBS for 5 min. After washing with PBS and blocking with 3% bovine serum albumin (BSA) in PBS for 30 min, the cells were incubated with monoclonal antibody (mAb) against AKAV N, 5F11 [24] for 1 h, followed by incubation with Alexa 594-conjugated anti-mouse IgGs (Jackson ImmunoResearch, West Grove, PA, USA) for 1 h. The cells were washed with PBS, dried, and mounted onto a Fluorescent Mounting Medium (Dako, Agilent, Santa Clara, CA, USA) with coverslips. Slides were stored in the dark at 4 °C until analysis. Fluorescence images were acquired using a Zeiss LSM700 confocal microscope. The total number of anti-AKAV antibody-stained areas was counted and the number of fluorescent focus was compared between the viruses. Data were collected from at least 4 wells for each virus.

### 2.7. Northern Blotting

The subconfluent HmLu-1 cell monolayer was infected with eGFP/38-AKAV or eGFP-AKAV at a MOI of 0.1, and the total cellular RNA was extracted every 12 to 72 h post-infection (hpi) using ISOGEN (NIPPON GENE, Tokyo, Japan). One microgram (1 μg) of the RNA was electrophoresed through an agarose gel (Reliant Gel system, Lonza Japan, Tokyo, Japan) in MOPS buffer and then transferred to a positively charged nylon membrane (Zeta-Probe Blotting Membranes, BIO-RAD, Hercules, CA, USA). The membrane was hybridized with biotinylated RNA probes. Detection was carried out using a VECTASTAIN Elite ABC Kit (VECTOR Laboratories, Burlingame, CA, USA) and Chemi-Lumi One super (Nacalai Tesque, Kyoto, Japan). The hybridized membrane images were obtained using LAS4000 mini (Fujifilm, Tokyo, Japan).

The biotinylated RNA probes, which recognized genome sense (-) or antigenome sense (+) sequences of eGFP or N/NSs regions, were prepared using PCR-amplified DNA fragment with a T7 promoter sequence as template and MEGAscript^®^ T7 Transcription Kit (Ambion, Thermo Fisher Scientific) with biotin-16-UTP (Roche, Basel, Switzerland), according to the manufacturer’s instructions. The probes were purified using a RNeasy Mini Kit (Qiagen).

### 2.8. Quantitative Real-Time PCR

Total cellular RNA used for northern blotting was also used for real-time PCR. Samples were polyadenylated in vitro using an A-Plus Poly(A) polymerase tailing kit (Epicenter Biotechnologies, Madison, WI, USA), according to the manufacturer’s instructions, and were subsequently purified using QIAamp viral RNA Mini Kit. The Poly(A) polymerase-treated RNAs (500 ng) were reverse-transcribed with a tag sequence containing oligo-dT primer (3′ RACE-AP; Invitrogen) using the PrimeScript RT Reagent Kit (Takara Bio, Shiga, Japan). The cDNAs were subjected to real-time PCR amplification with KOD SYBR qPCR Mix (TOYOBO) using primers homologous to the tag region of the oligo-dT primer (AUAP; Invitrogen) and GFP-qPCR_1, a primer specific for eGFP (5′- ACA TGG TCC TGC TGG AGT TC -3′) (Figure 4C). Similarly, 500 ng of Poly(A) polymerase-treated RNA were used to synthesize cDNA using oligo dT primer and random 6-mers, and the 18S rRNA primer pair (18S-qF and 18S-qR; sequences are provided upon request) was used to amplify an endogenous control gene. The reaction contained 10 µL of KOD SYBR qPCR Mix (TOYOBO), 1 µL of the RT reaction products, 6 (for GFP mRNA) or 4 pmol (for 18S rRNA) of each primer, 0.4 µL of 50X ROX reference dye, and water to a final volume of 20 µL. The reaction was performed in an ABI StepOnePlus with the following reaction conditions: 98 °C for 2 min, then 40 cycles of 98 °C for 10 s, 60 °C for 10 s, and 68 °C for 30 s. eGFP mRNA expression was quantitatively analyzed using the comparative CT method (∆∆CT) with the ABI StepOnePlus SDS software.

### 2.9. Animal Experiments with Bioimaging and Histopathological Analyses

Animal experiments were carried out with the approval of the University of Tokyo under the guidelines for animal and recombinant DNA experiments (approval number P15-051). Eight 3-day-old BALB/cCrSlc mice (Japan SLC, Shizuoka, Japan) were administered intraperitoneally with 1 × 10^5^ PFU per 0.1 mL dose of eGFP/38-AKAV, eGFP-AKAV, or AKAV-RG, or 0.1 mL of serum-free medium as a control, respectively. Mice were monitored daily for signs of disease and mortality for a period of 21 days. Animals that exhibited severe clinical symptoms or signs of moribund during the observation period were euthanized, and their brains, spinal cords, and the organs of the thorax and abdomen were extracted and observed under a LEICA MZ 10F microscope.

Images were acquired using a Nikon Coolpix P7000 digital camera or a Leica DMC6200 camera. For each imaging session, a mock-infected mouse inoculated with a serum-free medium was used as a negative control. Organs were then collected for histological examination and immunostaining.

For the detection of AKAV antigens, tissue samples including the brains were fixed with 4% phosphate-buffered paraformaldehyde and processed for paraffin embedding. Serial paraffin sections of 4 µm thickness were subjected to immunostaining using the Envision polymer method (Dako). Rabbit antiserum against the AKAV OBE-1 strain [25] (1:200 dilution) was used as the primary antibody [26], followed by reaction with the Dako Envision+ system horseradish peroxidase-labeled polymer anti-rabbit secondary antibody. Immunoreactivity was visualized with 0.05% 3,3′-diaminobenzidine plus 0.03% H_2_O_2_ in a Tris-hydrochloric acid buffer and then counterstained with hematoxylin.

## 3. Results

### 3.1. Generation of Recombinant Reporter AKAVs with 5′ UTR Partial Deletions of S Segment

We previously generated a recombinant reporter eGFP-AKAV using an ambisense strategy with an artificial S genome [12]. Although the eGFP-AKAV was generally capable of detecting infected cells in in vivo histopathological study, the fluorescence signal was too weak to apply to an in vivo imaging study. Here, we constructed a series of 5′ UTR partial deletion derivatives of the S-eGFP RNA plasmid (Figure 1A). Using these constructs, we attempted to rescue recombinant viruses by reverse genetics. All recombinant viruses possessing the S-eGFP deletion derivatives were rescued, except the S-eGFP/25 virus. The eGFP/42-, eGFP/41-, eGFP/40-, eGFP/39-, and eGFP/38-AKAVs were successfully generated without any insertions or mutations. Interestingly, 7–12 nt insertions, including the 3′-TTATAGT-5′ sequence, were compensated at deleted regions in eGFP/34-, eGFP/35-, eGFP/36-, and eGFP/37-AKAV. Such insertions occurred commonly in three independent reverse genetics experiments.

We selected eGFP/38- and eGFP/42-AKAVs for further analyses. To compare eGFP expression and CPE appearance between the viruses, we inoculated eGFP-AKAV, eGFP/38-AKAV, eGFP/42-AKAV, and wt AKAV viruses into HmLu-1 cells and observed these cells for two days (Figure 1B). As previously reported [12], eGFP expression of eGFP-AKAV was observed late after CPE appearance. By contrast, both 5′ UTR deletion viruses-infected cells expressed detectable levels of eGFP on 1 dpi without the clear appearance of CPE. Despite the same amounts of viruses used for inoculation, the eGFP/38-AKAV exhibited higher eGFP intensity than the eGFP/42-AKAV. These data indicated that the partial 5′ UTR deletions of ambisense S segment appreciably enhanced the eGFP expression in cell cultures.

To confirm the genetic stability of eGFP/42- and eGFP/38-AKAVs, we serially passaged them 10 times in HmLu-1 cells and then counted the number of plaques with passage 1 (P1), P5, P8, and P10 viruses. The ratios of eGFP-positive to total plaques were calculated (Figure 1C). The ratio of the original eGFP-AKAV was found to be genetically stable, as previously reported [12]. The GFP-positive ratios with eGFP/42-AKAV ranged from 78.4% to 85.9%, with a significant reduction at P8 and P10, suggesting the lower genetic stability of this virus. By contrast, the ratios of eGFP-positive plaques with eGFP/38-AKAV were 100% at P1–P8 and 97.5% at P10, indicating a robust genetic stability compared to eGFP/42-AKAV.

Collectively, we conclude that the eGFP/38-AKAV is the best candidate as the reporter AKAV for in vivo imaging.

### 3.2. In Vitro Growth Property of eGFP/38-AKAV

**Figure 2:**
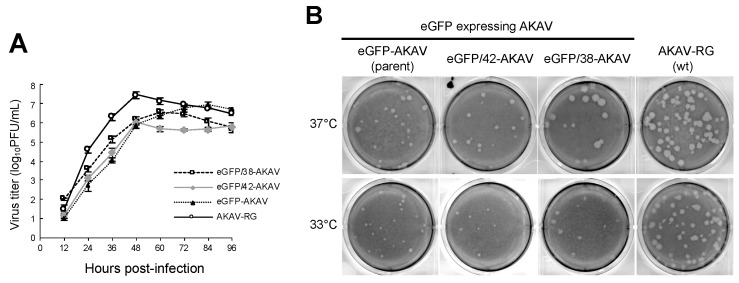
Growth kinetics and plaque morphology of recombinant viruses. (**A**) To assess the growth curves for recombinant viruses, HmLu-1 cells were infected with parent eGFP-AKAV, deletion viruses (eGFP/42- and eGFP/38-AKAVs), or wt AKAV-RG at a multiplicity of infection (MOI) of 0.01. The viral titers were determined using a plaque assay. The results are presented as the mean ± SD (error bars) from three independent experiments. (**B**) The plaque phenotypes of eGFP-, eGFP/42-, eGFP/38-AKAVs, and wt AKAV-RG viruses at 37 °C and 33 °C are shown.

To investigate the growth properties of the eGFP/38-AKAV in cell culture, we inoculated eGFP/38-AKAV, eGFP/42-AKAV, eGFP-AKAV, or wt AKAV into HmLu-1 cells at a MOI of 0.01 and determined their growth kinetics (Figure 2A). Although the titers of all reporter eGFP-AKAVs were 5–10 times lower than those of wt AKAV at most timepoints, the maximum titers (3.0 × 10^7^ PFU/mL at 48 hpi for wt AKAV, 3.5 × 10^6^ PFU/mL at 60 hpi for eGFP/38-AKAV, 1.1 × 10^6^ PFU/mL at 48 hpi for eGFP/42-AKAV, and 8.7 × 10^6^ PFU/mL at 84 hpi for eGFP-AKAV) were not significantly different between these viruses. The plaque sizes of eGFP/38-AKAV were similar to those of the wt virus; however, they were visibly larger than those of the parent eGFP-AKAV or eGFP-AKAV/42 at 37 °C (Figure 2B).

Since ambisense BUNVs was found to grow better at 33 °C than 37 °C in a previous report [13], forming larger plaques at 33 °C, unlike wt BUNV that formed plaques with similar sizes at both temperatures, we compared the plaque sizes of the eGFP-AKAVs at 33 °C and 37 °C. Although the plaque sizes of wt AKAV were similar at both temperatures, those of eGFP-AKAVs were smaller at 33 °C than 37 °C (Figure 2B), suggesting that the temperature-sensitivity of eGFP-AKAVs was different from that of ambisense BUNVs.

**Figure 3:**
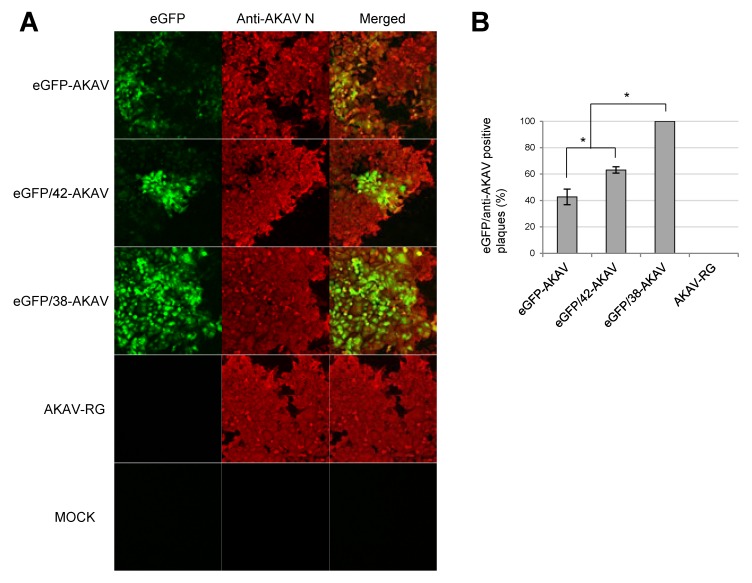
GFP expression of recombinant viruses. (**A**) HmLu-1 cells were infected with parent eGFP-AKAV, deletion viruses (eGFP/42- and eGFP/38-AKAVs), or wt AKAV-RG at a MOI of 0.01, or mock-infected. At 3 dpi, fluorescent microscopic images for the infected cells were obtained. The left panels show eGFP fluorescence (green) in cells infected with each virus or mock-infected. The middle panels show virus-infected cells detected with an anti-AKAV N mAb (red). The right panels indicate merged images with co-localization of eGFP and AKAV antigens. Magnification, ×100. (**B**) The ratios of numbers of eGFP-positive plaques to AKAV plaques were quantified. The results are presented as the mean ± SD (error bars) from 4–5 wells. *, *p* < 0.05, **, *p* < 0.01; Mann–Whitney U test.

To compare eGFP expression levels of the eGFP-AKAVs, we inoculated eGFP/38-AKAV, eGFP/42-AKAV, eGFP-AKAV, or wt AKAV into HmLu-1 cells. We tested eGFP signals and AKAV N antigens in infected cells at 3 dpi. The number and intensity of eGFP-positive cells were much higher in eGFP/38-AKAV-infected cells than in eGFP-AKAV as well as eGFP/42-AKAV-infected cells (Figure 3A). We also calculated the ratios of eGFP-positive plaques to total AKAV plaques, showing a significantly high ratio in the eGFP/38-AKAV-inoculated cells compared to the ratios in the eGFP-AKAV/42- and eGFP-AKAV-inoculated cells (Figure 3B). These data suggest that eGFP/38-AKAV possesses the preferred growth characteristics for its utility as a reporter AKAV.

### 3.3. Replication and Transcription of Ambisense S Segment RNA in Recombinant Virus-Infected Cells

**Figure 4:**
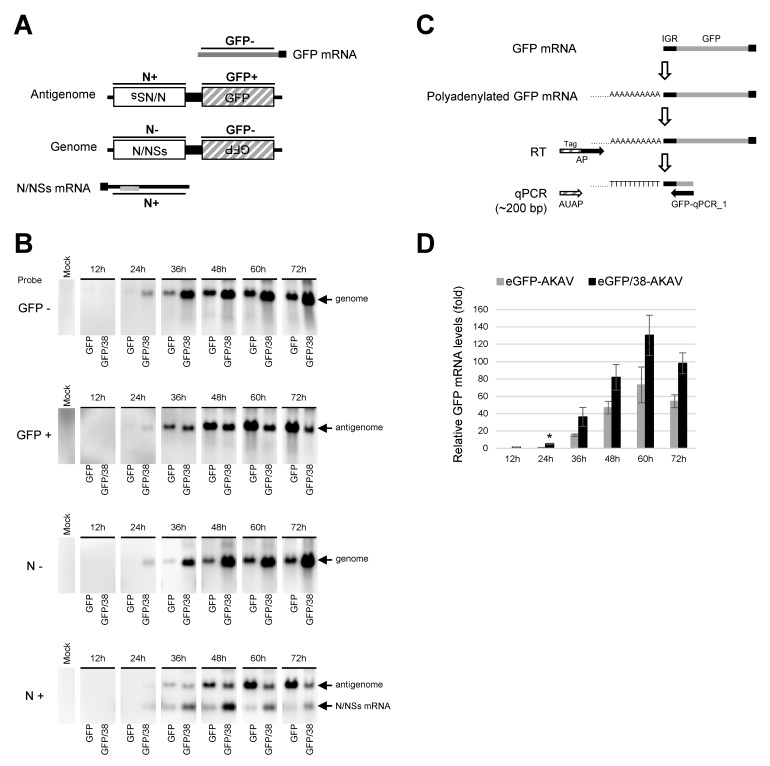
Analysis of virus-related RNAs in cells infected with recombinant viruses. (**A**) Schematic diagram of RNA probes used is shown as N (-), N (+), GFP (-), and GFP (+). (**B**) HmLu-1 cells were infected with either eGFP-AKAV (GFP) or eGFP/38-AKAV (GFP/38) at a MOI of 0.1. Total RNAs were extracted from the infected cells at 12 h intervals. Bands corresponding to genome RNA, antigenome RNA, and N/NSs mRNA were indicated by northern blot analysis using specific probes. (**C**) A qRT-PCR strategy was used to quantify GFP mRNA. Polyadenylated GFP mRNA was reverse-transcribed using AP primer (oligo dT primers containing tag sequence). The RT product was subjected to qPCR using AUAP primer (binding to the tag sequence) and GFP-qPCR_1 primer (binding to eGFP). (**D**) The qRT-PCR results are normalized to the level of eGFP mRNA extracted from cells infected with eGFP-AKAV at 24 hpi. The result with eGFP-AKAV at 12 hpi was excluded due to a distinct peak of melt curve. The results are presented as the mean ± SD (error bars) from three independent experiments. * *p* < 0.05; Mann–Whitney *U* test.

To examine the replicational and transcriptional modes of the deletion S RNA in virus-infected cells, we performed northern blotting for the RNAs collected from eGFP-AKAV- or eGFP/38-AKAV-infected cells at 12 to 72 hpi. Biotinylated probes were designed for the detection of viral specific RNA species: probe GFP- was used for the viral S RNA genome as well as eGFP mRNA; probe GFP+ was used for the antigenomic S RNA; probe N– was used for the viral S RNA genome; and probe N+ was used for the antigenomic S RNA and N/NSs mRNA (Figure 4A). The genomic S RNAs of eGFP-AKAV and eGFP/38-AKAV with probe GFP- or N– increased in time-dependent manners and the amounts of RNAs were higher with eGFP/38-AKAV than with eGFP-AKAV at every time point (Figure 4B). By contrast, antigenomic S RNAs with probe GFP+ or N+ were lower with eGFP/38-AKAV than eGFP-AKAV, especially in the later time-points. Interestingly, antigenomic S RNAs with eGFP/38-AKAV decreased at 72 hpi unlike with eGFP-AKAV. The partial 5′ UTR deletion of S RNA may have decreased the synthesis of antigenomic RNA from genomic RNA owing to qualitative alteration of promoter structure in the UTRs or may have simply affected the stability of the antigenomic RNA. The deletion in the N/NSs mRNAs with probe N+ were higher with eGFP/38-AKAV than eGFP-AKAV. No eGFP mRNA with probe GFP- was detected in the northern blotting, suggesting an undetectable expression level of eGFP mRNAs.

Therefore, we determined the amounts of eGFP mRNAs by qRT-PCR, instead of northern blotting. Since viral S genome and eGFP mRNA have the same orientation, they cannot be distinguished with a primer binding to eGFP ORF region. Therefore, we added poly-A and tag sequences to the 3′-end of eGFP mRNA derived from eGFP/38-AKAV by using poly-A polymerase and reverse-transcribed the mRNA with tag sequence containing oligo dT primer. Then, we performed qPCR using the primers binding to the tag and eGFP ORF (Figure 4C). Following qPCR testing, we confirmed the size of the RT-PCR products by agarose gel electrophoresis and detected only a band corresponding to the eGFP mRNAs (~200 bp) but not to full-length S genome (~1 kbp). The eGFP mRNA reached a peak at 60 hpi both in eGFP/38-AKAV- and eGFP-AKAV-infected cells. The mRNA levels were significantly higher in eGFP/38-AKAV- than in eGFP-AKAV-infected cells at most time points (Figure 4D).

Taken together, these data suggest that the deletion of S RNA 5′ UTR with eGFP/38-AKAV enhanced eGFP mRNA transcription and altered its RNA replication mode, resulting in the inefficient transcription of antigenome RNA from S RNA genome template, compared to the parent eGFP-AKAV.

### 3.4. Pathogenicity of eGFP/38-AKAV in Mice

**Figure 5:**
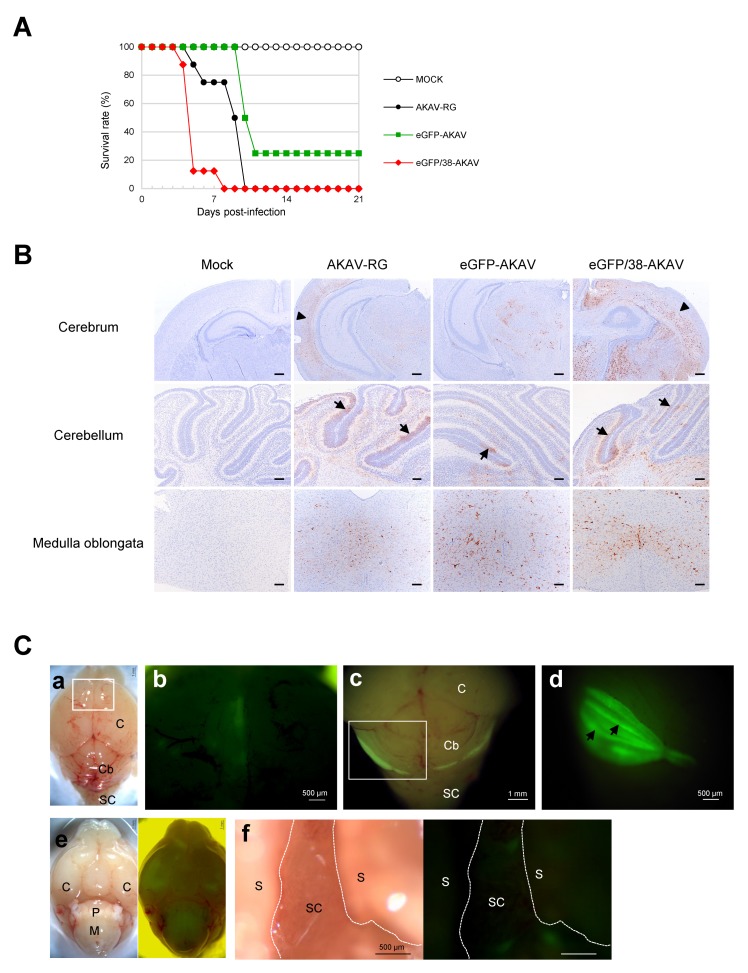
Experimental infection of mice with recombinant viruses. Suckling mice were intraperitoneally infected with AKAV-RG, eGFP-AKAV, or eGFP/38-AKAV, or mock-infected and monitored for 3 weeks. (**A**) Survival curves of mice after infection with 1 × 10^5^ PFU of each virus are shown. (**B**) Paraffin sections of the central nervous system (CNS) tissues, such as cerebrum, cerebellum, and brainstem, including medulla oblongata, from each virus or mock-infected mice were immunostained for AKAV antigens using anti-AKAV polyclonal antibody. Viral antigens are shown in brown. The arrowheads in the cerebrum indicate virus-infected neurons in the cerebral cortex. The arrows in the cerebellum indicate virus-infected cells inside of the external granular layer in the cerebellum, corresponding to a cerebellar fissure anatomically. Bar, 100µm. (**C**) Macroscopic fluorescence in the CNS of mice infected with eGFP/38-AKAV is shown. Viral infection was detected in the cerebrum (**a** and **b**, 7 dpi), cerebellum (**c** and **d**, 5 dpi), brainstem (**e**, 8 dpi), and lumbar spinal cord (**f**, 8 dpi). (**b**) Higher magnification of the box area in panel (**a**) light field is shown. Panel (**d**) is the caudal aspect of the cerebellum, indicated by a square in panel (**c**) at higher magnification. Intensive infection of eGFP/38-AKAV was detected in the cerebellum, in particular cerebellar fissure, as indicated by arrows. (**e**) Light field (left) and eGFP (right) images of bottom aspect are shown. C, cerebrum; Cb, cerebellum; P, pons; M, medulla oblongata; S, spine; SC, spinal cord.

To determine the pathogenicity of eGFP/38-AKAV, we intraperitoneally inoculated 1 × 10^5^ PFU of eGFP/38-AKAV, eGFP-AKAV, or wt AKAV into three-day-old suckling mice and observed their clinical symptoms and survival rates (Figure 5A). All mice inoculated with wt AKAV represented neurological signs, such as tremor, convulsion, paralysis, and astasia, and died before 10 dpi. Similarly, both eGFP/38-AKAV- and eGFP-AKAV-infected mice showed severe neurological symptoms and became moribund. eGFP/38-AKAV exhibited a 100% mortality rate, as with wt AKAV, however, the mice were killed at earlier time-points compared to wt AKAV. eGFP-AKAV did not kill all the mice. These data suggest that eGFP/38-AKAV possessed higher pathogenicity than wt AKAV and eGFP-AKAV in mice.

To detect the precise distribution of viral antigens in eGFP/38-AKAV-infected mice, we subjected the tissues of the moribund mice to histopathological analysis. No remarkable gross changes were observed in any organs of virus-infected mice. However, the brains of some mice were found to develop cerebral malacia, presumably due to necrosis. Microscopically, necrosis and inflammation were observed particularly in the brains of eGFP/38-AKAV- and wt AKAV-infected mice. Characteristic lesions were perivascular infiltration of white blood cells in the cerebral cortex and, more severely, in the brainstem, including the pons and medulla oblongata. Karyopyknosis was also observed in the outside nuclear layer of the cerebellum. Viral antigens were detected in the cerebrum, cerebellum, and brainstem, including the medulla oblongata, in all the virus-infected groups (Figure 5B). A remarkable amount of viral antigen-positive cells was observed in the brain of the eGFP/38-AKAV- and wt AKAV-infected mice, and the infected cell distributions with these two viruses were very similar. eGFP/38-AKAV and wt AKAV preferentially infected in neurons in the deep cortical layers rather than the surface of the cerebral cortex (Figure 5B, arrowheads). In addition, AKAV positive cells were observed mainly inside the external granule layer (EGL) of the cerebellar cortex, where they were located at the cerebellar fissure, in eGFP/38-AKAV- and wt AKAV-infected mice (Figure 5B, arrows). EGL is observed on the surface of the cerebellar hemisphere in the early postnatal stages of the mice. Mitotically active and postmitotic granule cell precursors (GCPs) are located within the EGL. Postmitotic GCPs migrate to the cerebellar cortex during development. Finally, they localize to the mature inner granular layer, where the EGL dissolves. Although the outside region of the cerebellum cortex, close to the pia mater, seemed to undergo apoptosis, only a few antigen positive cells were observed. These data suggest that eGFP/38-AKAV possesses a pathogenicity and in vivo dynamics that are similar to wt AKAV.

### 3.5. Fluorescence Imaging of the Central Nervous System (CNS) in eGFP/38-AKAV-Infected Mice

To visualize the spread of the virus, we subjected eGFP/38-AKAV-infected mice to macroscopic fluorescence imaging using fluorescent stereoscopic microscopy. At the moribund stage (5–8 dpi), fluorescence was observed in the cerebral hemisphere, cerebellum, pons, and medulla oblongata (Figure 5C(a–e)). The highest intensity of fluorescence was observed in cerebellar fissure of cerebellar hemisphere and vermis rather than the cerebellar folia (Figure 5C(c,d)). In addition, we incised the spinal cord and observed GFP-positive foci at the lumbar spinal cord (Figure 5C(f)). GFP signals were neither observed in the intrathoracic organs nor in the intraabdominal organs. The fluorescent distributions corresponded to results of the immunohistochemical analysis. No fluorescence was observed in any of the eGFP-AKAV-, AKAV-RG-, and mock-infected mice tissues.

## 4. Discussion

In this study, we established an improved reporter AKAV-expressing eGFP (designated as eGFP/38-AKAV), whose intensity of expressed eGFP, genetic stability, and pathogenicity in mice was superior to our previous reporter, eGFP-AKAV. The eGFP/38-AKAV expressed sufficient levels of eGFP in the organs of immunocompetent mice, observed under a fluorescent stereoscopic microscope, suggesting that this reporter virus could be applied in further in vivo imaging studies for the analysis of the precise AKAV dynamics in animals, as has been previously conducted with several other viruses [27,28,29,30,31,32].

In previous reports using another orthobunyavirus, BUNV, the nucleotide deletion of a 5′ UTR internal region of the S RNA decreased the viral titers due to inefficiency of RNA replication and/or transcription [4,33]. On the contrary, our eGFP/38-AKAV enhanced viral proliferation with the same deletions, compared to the parent eGFP-AKAV. One plausible reason for this discrepancy is that the ambisense AKAV may not require the transcriptional termination signal at 5′ UTR of the S RNA for gene expression. Here, we showed that the deletion of the 5′ UTR internal region of S RNA enhanced the transcription of eGFP mRNA, as well as synthesis of genomic S RNA from an antigenomic S RNA template (Figure 4). Although the function of the 5′ UTR internal region of AKAV S RNA is not known, the destruction of the loop structure at the transcriptional termination signal [18] may allow for viral polymerase extension. By contrast, although the amount of genomic S RNA of eGFP/38-AKAV was found to increase in the infected cells, the amount of its antigenomic S RNA was lower, in particular at the later time-points of infection, compared to eGFP-AKAV. However, the transcription of N/NSs mRNA from the genomic RNA template did not seem to be affected; the amount of N/NSs mRNA was higher than that of eGFP-AKAV at any time point. Interestingly, we found that larger deletions of the 5′ UTR sequence caused an insertion containing a fixed sequence in its deleted region (Figure 1A), suggesting that the insertion is required for genome replication. Taken together, these results indicate that the replication and transcription of ambisense AKAV S RNA are regulated by viral polymerase promoter and/or terminator activities, determined by the length and arrangement of the 5′ UTR sequences, which affect the interactions between the 3′ and 5′ UTR sequences. This will need to be studied further to fully clarify this point.

Interestingly, eGFP/38-AKAV is more virulent than wild-type AKAV in mice (Figure 5A). Although the mechanism for this observation is unknown, there is a possible difference in the replication properties in brain tissues of eGFP/38-AKAV- and wild-type AKAV-infected mice. Indeed, more viral antigens were detected in brain tissues of the mutant virus- than that of wild-type virus-infected mice (Figure 5B). Further studies are now in progress to clarify this observation.

Microscopic imaging assessment was used to determine the location and distribution of eGFP/38-AKAV, which was visualized in the brain and spinal cord, the main known sites of viral proliferation in the natural host [34,35,36,37,38]. Although AKAV antigens in the cerebrum and brainstem showed diffuse distribution, except for at the surface layer in all infected groups, by immunostaining (Figure 5B), we successfully detected sufficient fluorescence in such tissues under the stereoscopic microscope (Figure 5C). We observed the consistent localization of immunostaining-positive GCPs in the EGL of the brains in the mice infected with the mutant as well as the wild-type viruses. We also found that the GCPs of the superficial EGL of the cerebellum had already undergone apoptosis at the moribund stage in the mice infected with both the viruses, and that rapid disease progression may account for the few AKAV positive cells found in the area. Although the presence of fetal or postnatal cattle EGL in the cerebellum has not yet been studied, AKAV could infect these regions in naturally-infected cattle and result in neurological diseases. Taken together, eGFP/38-AKAV possessed similar pathogenicity as the wild-type virus. Hence, this recombinant virus could be utilized for in vivo cattle studies in the future.

In conclusion, our reporter eGFP/38-AKAV will allow researchers to directly monitor AKAV infection and disease progression in the infected host to elucidate pathogenesis of AKAV in animals at cellular levels in organs using imaging technologies, in combination with other detection methods such as immunostaining for viral proteins. Application of this strategy in other non-neurovirulent AKAV strains may also contribute to such studies. Furthermore, the same strategy could be adopted to generate recombinant orthobunyaviruses with foreign reporter genes in their S segments, which provides a new opportunity to understand the dynamics of viruses with their infected host.
